# Inhibitory Effect of mTOR Activator MHY1485 on Autophagy: Suppression of Lysosomal Fusion

**DOI:** 10.1371/journal.pone.0043418

**Published:** 2012-08-22

**Authors:** Yeon Ja Choi, Yun Jung Park, Ji Young Park, Hyoung Oh Jeong, Dae Hyun Kim, Young Mi Ha, Ji Min Kim, Yu Min Song, Hyoung-Sam Heo, Byung Pal Yu, Pusoon Chun, Hyung Ryong Moon, Hae Young Chung

**Affiliations:** 1 Molecular Inflammation Research Center for Aging Intervention (MRCA), College of Pharmacy, Pusan National University, Busan, Korea; 2 Laboratory of Medicinal Chemistry, College of Pharmacy, Pusan National University, Busan, Korea; 3 Plant Systems Engineering Center, KRIBB, Daejeon, Korea; 4 Department of Physiology, The University of Texas Health Science Center at San Antonio, San Antonio, Texas, United States of America; 5 College of Pharmacy, Inje University, Gimhae, Gyeongnam, Korea; University of Quebect at Trois-Rivieres, Canada

## Abstract

Autophagy is a major degradative process responsible for the disposal of cytoplasmic proteins and dysfunctional organelles via the lysosomal pathway. During the autophagic process, cells form double-membraned vesicles called autophagosomes that sequester disposable materials in the cytoplasm and finally fuse with lysosomes. In the present study, we investigated the inhibition of autophagy by a synthesized compound, MHY1485, in a culture system by using Ac2F rat hepatocytes. Autophagic flux was measured to evaluate the autophagic activity. Autophagosomes were visualized in Ac2F cells transfected with AdGFP-LC3 by live-cell confocal microscopy. In addition, activity of mTOR, a major regulatory protein of autophagy, was assessed by western blot and docking simulation using AutoDock 4.2. In the result, treatment with MHY1485 suppressed the basal autophagic flux, and this inhibitory effect was clearly confirmed in cells under starvation, a strong physiological inducer of autophagy. The levels of p62 and beclin-1 did not show significant change after treatment with MHY1485. Decreased co-localization of autophagosomes and lysosomes in confocal microscopic images revealed the inhibitory effect of MHY1485 on lysosomal fusion during starvation-induced autophagy. These effects of MHY1485 led to the accumulation of LC3II and enlargement of the autophagosomes in a dose- and time- dependent manner. Furthermore, MHY1485 induced mTOR activation and correspondingly showed a higher docking score than PP242, a well-known ATP-competitive mTOR inhibitor, in docking simulation. In conclusion, MHY1485 has an inhibitory effect on the autophagic process by inhibition of fusion between autophagosomes and lysosomes leading to the accumulation of LC3II protein and enlarged autophagosomes. MHY1485 also induces mTOR activity, providing a possibility for another regulatory mechanism of autophagy by the MHY compound. The significance of this study is the finding of a novel inhibitor of autophagy with an mTOR activating effect.

## Introduction

Autophagy is a cellular process responsible for the degradation of cytoplasmic components via the lysosomal pathway [1], [2]. As an essential cellular housekeeping system, autophagy occurs at low baseline levels in all cells and contributes to maintaining cellular homeostasis [3], [4]. Autophagy is upregulated beyond basal levels when cells need to utilize intracellular nutrients under certain conditions such as starvation, hypoxia, and growth factor withdrawal [5], [6]. During the autophagic process, cells form double-membraned vesicles called autophagosomes that sequester disposable materials in the cytoplasm. Autophagosomes fuse with lysosomes to form autolysosomes, and then the sequestered contents undergo degradation by lysosomal hydrolases [7].

During the formation of the autophagosome cytosolic microtubule-associated protein 1 light chain 3-I (LC3I) is cleaved and conjugated with phosphatidylethanolamine (PE), leading to formation of LC3II, an autophagic vacuole-associated form [8]. Thus, an increase in the amount of the LC3II protein and an increase in the LC3II/LC3I ratio have been considered hallmarks of autophagy. However, LC3II of the inner membrane of the autophagosome is also degraded by lysosomal proteases after the formation of autolysosomes, and the extent of autophagosome formation often dissociates from the level of autophagic flux. Therefore, the static LC3II/LC3I ratio, which shows LC3I and LC3II at a single time point, can produce misleading results. For this reason, we measured autophagic flux to evaluate autophagic activity. Autophagic flux, which expresses autophagic activity and its nature as a dynamic process, is assessed by comparing the LC3II/LC3I ratio in the absence and presence of lysosomal inhibitors such as bafilomycin A1 and chloroquine. In addition, p62/SQSTM1 is a useful marker of autophagic activity. The scaffolding adaptor protein p62 interacts with both LC3II and polyubiquitinated protein, which leads to the self-degradation as well as degradation of polyubiquitinated proteins in autolysosomes. [9], [10], [11].

Autophagy is a complicated process that consists of autophagosome biogenesis, maturation, and fusion with lysosomes. A number of diverse signaling complexes have effects on the respective molecular steps in the regulation of the autophagic process [12], [13]. There are several pharmacological compounds, including rapamycin, a well-known specific inhibitor of mammalian target of rapamycin (mTOR), that have been found to induce the autophagic process [14]. Rapamycin has been used as a conventional reagent for autophagy activation since it was first discovered that its inhibition of mTOR strongly induced autophagy. On the other hands, a few inhibitors of autophagy including 3-methyladenine (3-MA), chloroquine, and bafilomycin A1, are employed in experimental and clinical research [15], [16], [17]. 3-MA inhibits class III phosphoinositide 3 kinase (PI3K) that positively regulates the formation of autophagosomes. Bafilomycin A1 and chloroquine, which are useful tools in assessment of autophagic flux mentioned above, inhibit the final step of autophagy. Bafilomycin A1, a macrolide antibiotic isolated from *Streptomyces griseus* strains, specifically inhibits vacuolar H^+^ ATPase (V-ATPase), which plays a role in the acidification of the lysosome leading to the digestive function of the lysosome [18]. Chloroquine also inhibits fusion between autophagosomes and lysosomes through unknown molecular targets.

**Figure 1:**
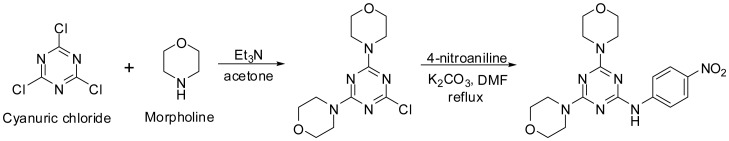
Brief scheme of the synthetic process of MHY1485.

Here, we have shown the effect of our synthesized 4,6-dimorpholino-*N*-(4-nitrophenyl)-1,3,5-triazin-2-amine (MHY1485) on the arrest of the autophagic process through the inhibition of lysosomal fusion in normal rat liver Ac2F cells. MHY1485 was synthesized based on its morpholino triazine structure that is known to bind mTOR (Fig. 1), and was selected based on the results of the ratio of LC3BII/LC3BI [19], [20]. However, the effect of MHY1485 on autophagic regulation was first studied in this study. In our result, LC3II protein largely accumulated and autophagosomes enlarged by the treatment of MHY1485. In addition, MHY1485 induced mTOR activity, which is another regulator of autophagy. We strongly suggest that the synthetic MHY compound has potential as a new inhibitor of autophagy.

## Materials and Methods

### Materials

The structure of synthesized MHY1485 is shown in Figure 1. All chemical reagents were obtained from Sigma (St. Louis, MO, USA), except where noted. Antibodies against LC3B, p62/SQSTM1, beclin-1, phospho-mTOR (Ser2448), mTOR, phospho-4E-BP1 (Thr37/46), and 4E-BP-1 were obtained from Cell Signaling (New England Biolabs, Hertfordshire, UK), and antibody against β-Actin, anti-rabbit IgG-horseradish peroxidase-conjugated secondary antibody, and anti-mouse IgG-horseradish peroxidase-conjugated secondary antibody were from Santa Cruz Biotechnology (Santa Cruz, CA, USA). LysoTracker® was from Molecular Probes, Inc. (Eugene, OR, USA). Polyvinylidene difluoride (PVDF) membranes were obtained from Millipore Corporation (Bedford, MA, USA). Westsave™ western blot detection kit was obtained from Young In Frontier Co. (Anyang, Korea). Dokdo-MARK™ protein size marker was obtained from ElpisBiotech (Taejeon, Korea). Sterile plasticware for tissue cultures was purchased from SPL Labware (Seoul, Korea). All other materials were obtained in the highest available grade.

**Figure 2:**
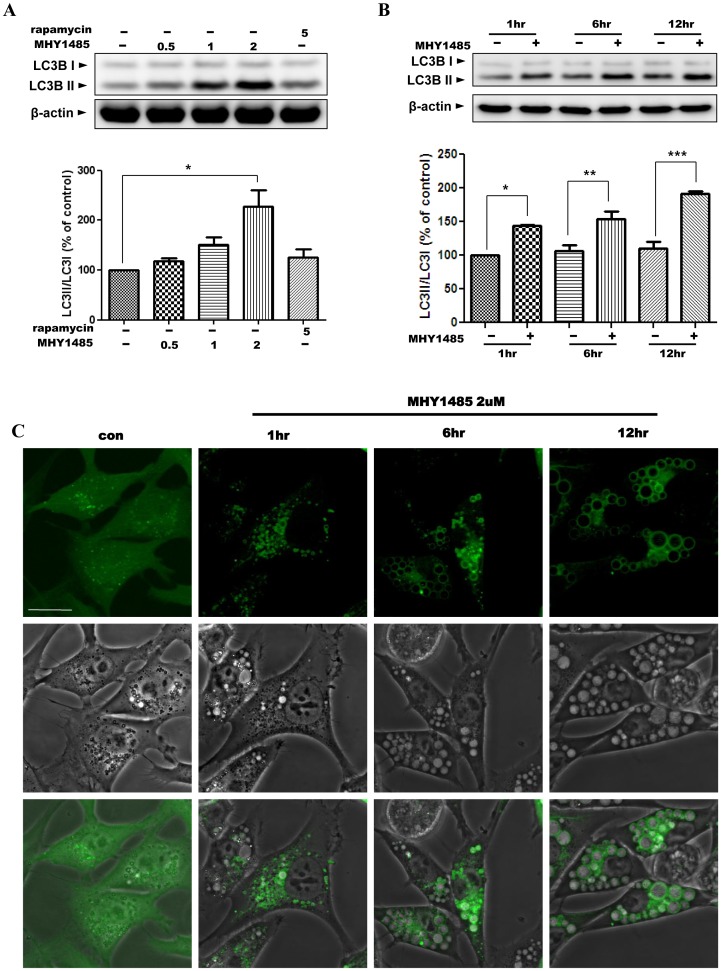
Increase of the LC3II/LC3I ratio and LC3II-positive vacuoles by MHY1485. Western blot analysis was performed to detect LC3II and LC3I. Ac2F cells were treated with different concentrations of MHY1485 and rapamycin 5 µM as a positive control for 6 h. Bars represent the LC3II/LC3I ratio calculated by normalizing the LC3II/LC3I ratio from MHY1485-treated or rapamycin-treated samples with the LC3II/LC3I ratios from untreated samples (A). Control cells treated with same volume of vehicle or MHY1485 (2 µM) for 1, 6 or 12 h were collected. Bars represent the LC3II/LC3I ratio calculated by normalizing the LC3II/LC3I ratio from MHY1485-treated samples with LC3II/LC3I ratio from control samples at 1 hour (B). β-Actin blot is shown to verify the same amount of protein loaded. The blots were quantified by densitometry expressed as mean±SD (*p<0.05, **p<0.01, ***p<0.001; n = 3). Live-cell confocal microscopic images of AdGFP-LC3-transfected Ac2F cells treated with 2 µM MHY1485 for 1, 6 or 12 h are shown. The images show the GFP-LC3-positive vacuoles (upper, green), corresponding phase contrast images (middle) and merged images (bottom). Scale bar, 20 µm.

### Synthesis and Chemical Property of MHY1485

All reagents were purchased from Aldrich and were used without any further purification. Melting points are uncorrected. Nuclear Magnetic Resonance (NMR) data were recorded on a Varian Unity INOVA 400 spectrometer and Varian Unity AS 500 spectrometer, using CDCl_3_ or DMSO-d_6_ and chemical shifts were reported in parts per million (ppm) with reference to the respective residual solvent or deuteriated peaks (_H_ 2.49 and _C_ 40.0 for DMSO-*d*_6_, _H_ 7.26 and _C_ 77.0 for CDCl_3_). Coupling constants are reported in hertz. The abbreviations used are as follows: s (singlet), d (doublet), m (multiplet). The electron-impact ionized mass spectra (EIMS) were obtained at an electron beam energy of 75 eV. All the reactions described below were performed under nitrogen or argon atmosphere and monitored by thin-layer chromatography (TLC). TLC was performed on Merck precoated 60 F_254_ plates. Column chromatography was performed using silica gel 60 (230–400 mesh, Merck). All anhydrous solvents were distilled over CaH_2_ or Na/benzophenone prior to use.

**Figure 3:**
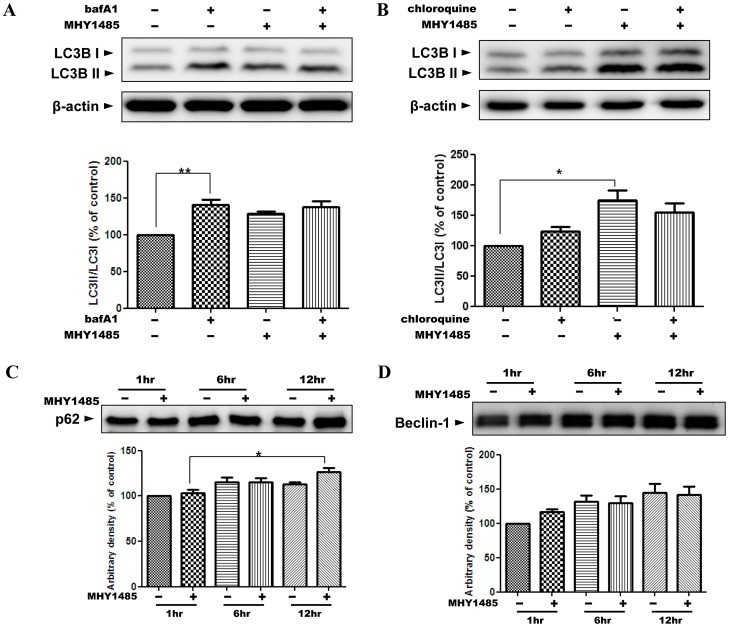
Failure of the increase of autophagic flux. Western blot analysis was performed with samples from cells treated in 2 µM MHY1485 for 6 h. The lysosomotropic agents bafilomycin A1 (bafA1, 10 nM) and chloroquine (100 µM) were applied 1 h before the cell harvest to measure the autophagic flux (A, B). Bars represent the LC3II/LC3I ratio normalized with the LC3II/LC3I ratio from untreated samples. β-Actin blot is shown to verify the same amount of protein loaded. The blots were quantified by densitometry expressed as mean±SD (**p<0.01; n = 3). C and D show the immunoblots of p62 and beclin-1 in samples from the cells treated with MHY1485 or the same amount of vehicle for different times. The blots were quantified by densitometry and were normalized with the control sample at 1 h expressed as mean±SD (*p<0.05; n = 3).

### 4,4′-(6-Chloro-1,3,5-triazine-2,4-diyl)dimorpholine

To a stirred solution of cyanuric chloride (5 g, 27.1 mmol) in acetone (30 mL) and crushed ice, a mixture of triethylamine (8.15 mL, 111 mmol) and morpholine (4.72 mL, 54.2 mmol) was added at −10°C. After the addition, the reaction mixture was stirred at room temperature for 1 h and diluted with 50 mL of water. The white solid generated was filtered and washed with water and acetone. The white solid was dried under reduced pressure (5.79 g, 74.9% yield). The final product precipitated from methanol washing needed no further purification as its purity was ascertained by TLC using a multiple developments method and ^1^H and ^13^C NMR experiments.

**Figure 4:**
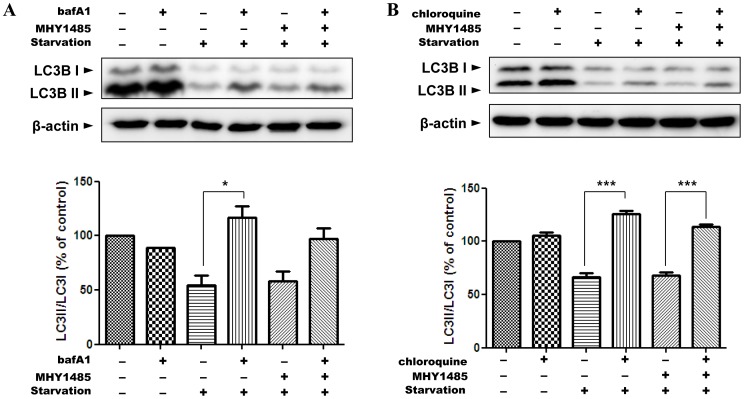
Inhibition of starvation-induced autophagic flux by MHY1485. Western blot analysis was performed to measure autophagic flux. Ac2F cells under starvation or co-treatment of 2 µM MHY1485 for 6 h were treated 1 h before the cell harvest with lysosomotropic agents bafilomycin A1 (10 nM) and chloroquine (100 µM) (A, B). Bars represent the LC3II/LC3I ratio normalized with the LC3II/LC3I ratio from untreated samples. β-Actin blot is shown to verify the same amount of protein loaded. The blots were quantified by densitometry expressed as mean±SD (*p<0.05, ***p<0.001; n = 3).

**Figure 5:**
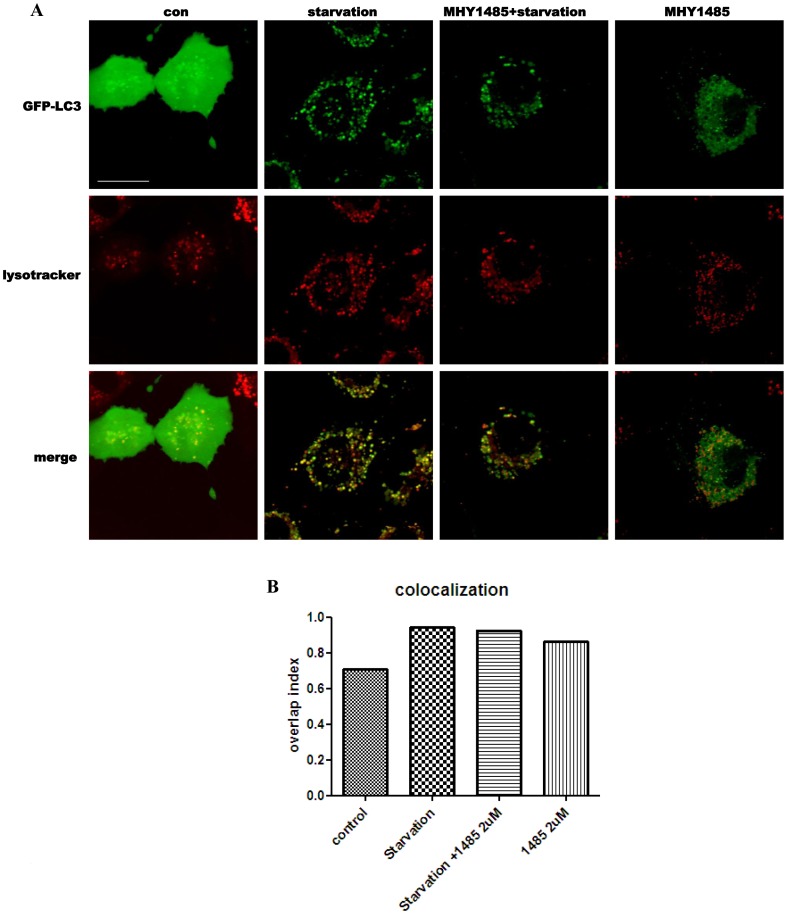
MHY1485 inhibition of starvation-induced colocalization between autophagosomes and lysosomes. Live-cell confocal microscopic images of AdGFP-LC3 (green) and lysosomes (red) stained by LysoTracker were collected from Ac2F cells under starvation or co-treatment of 2 µM MHY1485 for 6 h. Merged images show the co-localization of LC3-positive autophagosomes and lysosomes (yellow). Scale bar, 20 um. Bars represent the level of overlap index.

Melting point (m.p): 172.6–173.9°C; ^1^H NMR (400 MHz, CDCl_3_) δ 3.74–3.67 (m, 16 H, 8×CH_2_); ^13^C NMR (100 MHz, CDCl_3_) δ 169.8 (C2, C4), 164.7 (C6), 66.9 (4×OCH_2_), 44.1 (4×NCH_2_); IR (KBr): 1504, 1355, 1122, 806 cm^−1^; Mass (EIMS), 285 (M)^+^.

### 4,6-Dimorpholino-N-(4-nitrophenyl)-1,3,5-triazin-2-amine

A suspension of 4,4′-(6-chloro-1,3,5-triazine-2,4-diyl)dimorpholine (300 mg, 1.05 mmol), 4-nitroaniline (159.5 mg, 1.15 mmol), and potassium carbonate (159.5 mg, 1.15 mmol) in N,N-dimethylformamide (2 mL) was refluxed for 5 h. The precipitates generated were filtered on a Buchner funnel and washed methanol and water. After being dried under reduced pressure, the title product (258.6 mg, 63.6%) was obtained as a pale yellow solid.

m.p: 265.0–266.2°C; ^1^H NMR (500 MHz, CDCl_3_) δ 8.20 (d, 2 H, J = 9.0 Hz, 3′-H, 5′-H), 7.70 (d, 2 H, J = 9.0 Hz, 2′-H, 6′-H), 7.02 (s, 1 H, NH), 3.81–3.79 (m, 8 H, 2×CH_2_), 3.76–3.74 (m, 8 H, 4×CH_2_); ^13^C NMR (100 MHz, DMSO-d_6_) δ 165.2 (C4, C6), 164.6 (C2), 147.8 (C1′), 141.1 (C4′), 125.5 (C3′, C5′), 119.1 (C2′, C6′), 66.6 (4×OCH_2_), 44.1 (4×NCH_2_); IR (KBr): 3368, 1535, 1502, 1338, 1111, 851 cm^−1^.

**Figure 6:**
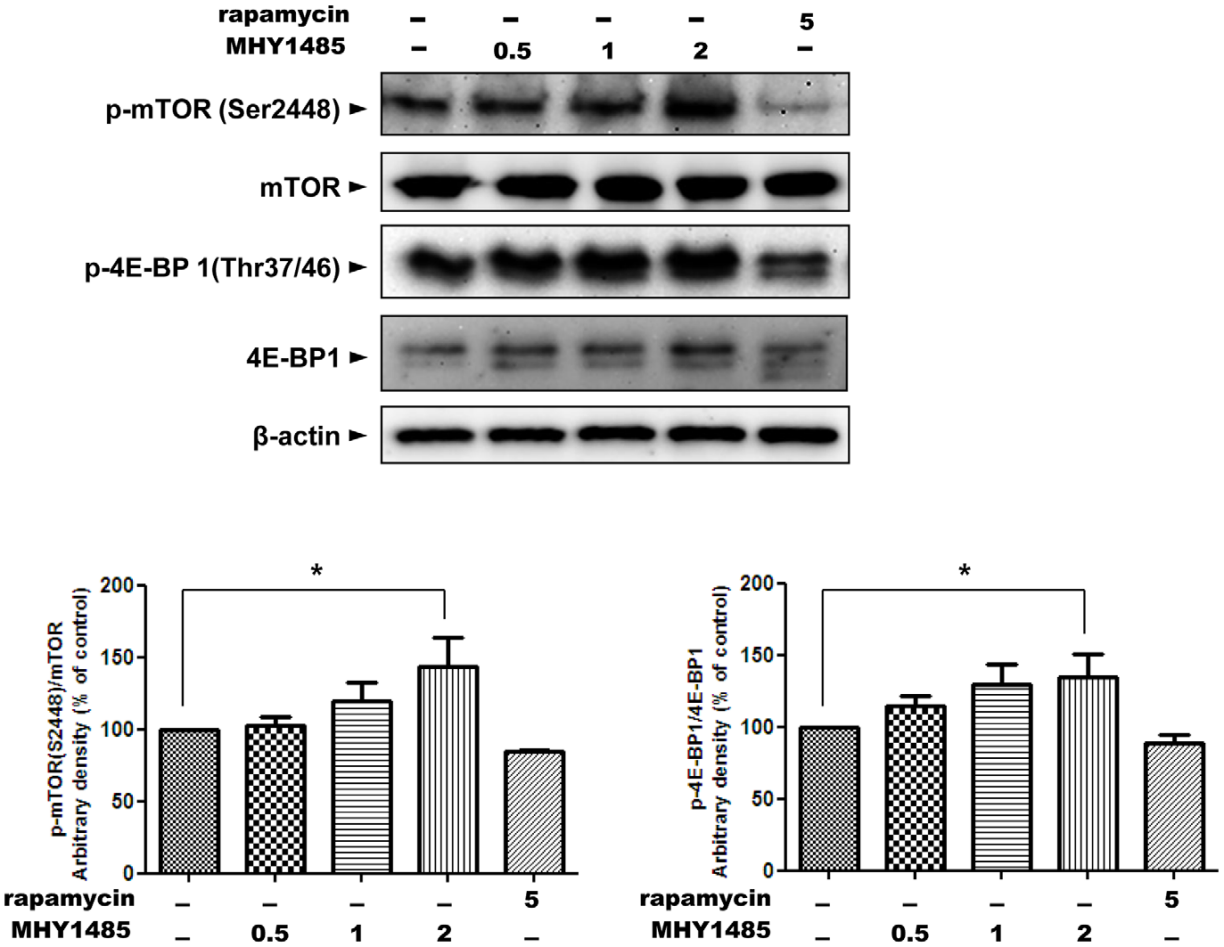
Activation of mTOR by MHY1485. Western blot analysis was performed to detect the change of total protein level and levels of phosphorylated forms of mTOR and 4E-BP1 reflecting the activity of mTOR. Ac2F cells were treated with MHY1485 of different concentrations and rapamycin 5 µM as a positive control for 1 h. Bars represent the phospho-mTOR(Ser2448)/mTOR ratio and the phospho-4E-BP1(Thr37/46)/4E-BP1 ratio normalized with the ratio from untreated samples, respectively. β-Actin blot is shown to verify the same amount of protein loaded. The blots were quantified by densitometry expressed as mean±SD (*p<0.05; n = 3).

**Figure 7:**
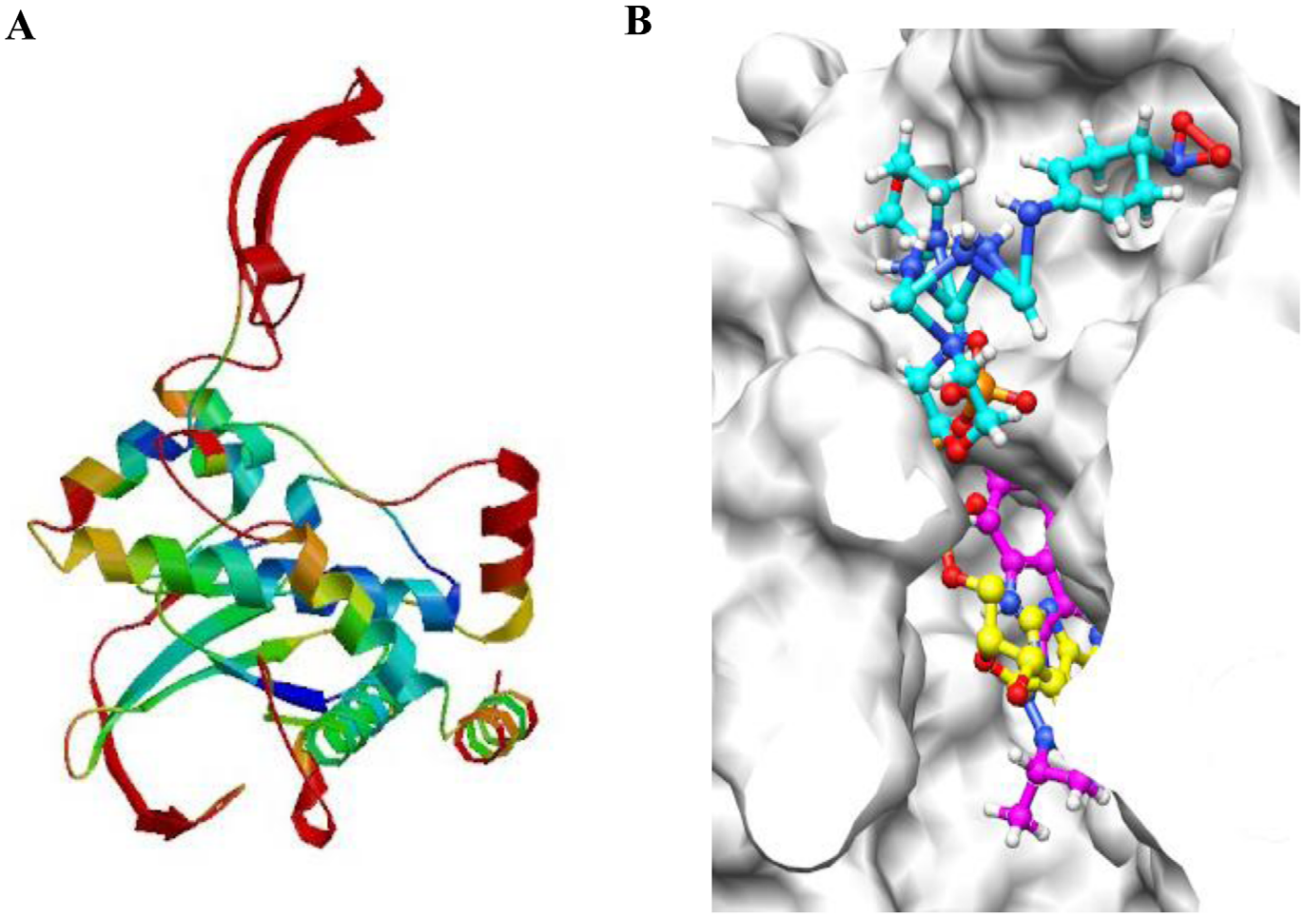
Three-dimensional model of mTOR and docking simulation between mTOR and PP242 and MHY1485. A three-dimensional model of mTOR (NCBI GI number: 4826730) was built using SWISS-MODEL program (A). Homologue structural template used was 1E8X (18% sequence identity and 6.9E-44 e-value). The Z-score of the predicted model was −4.8. We checked the quality of each residue on the mTOR structure model. The internal region, which bound ATP and target compounds, was more reliable (blue) than the external region (red) of mTOR. The estimated residue error was visualized using a color gradient from blue (more reliable regions) to red (potentially unreliable regions, estimated error above 3.5 Å). B shows the docking simulation between mTOR and PP242 and MHY1485. The magenta compound is PP242, which was used as a control compound. The cyan compound is MHY1485, and the yellow compound is ATP. The binding energies of the compounds were −7.28 kcal/mol (PP242) and −7.55 kcal/mol (MHY1485).

### Cell Culture System

Rat hepatocytes, Ac2F cells were obtained from ATCC (Manassas, VA, USA). The cells were grown in Dulbecco’s modified eagle medium (DMEM, HyClone Laboratories, Utah, USA) containing 2 mM l-glutamine, 100 units/ml penicillin, 100 µg/ml streptomycin, and 10% heat-inactivated fetal bovine serum (FBS, HyClone). Cells were maintained at 37°C in a humidified atmosphere containing 5% CO_2_/95% air. Under starvation conditions, cells were treated with Hank’s Balanced Salt Solution (HBSS) (GIBCO®, Grand Island, NY, USA). Our compound was treated under the same conditions as the growth medium to exclude the effect of serum reduction to induce autophagy. For assessment of autophagic flux, the lysosomotropic agents bafilomycin and chloroquine were applied 1 h before the cell harvest at concentrations of 10 nM and 100 µM, respectively [21], [22].

### Western Blotting

Cells were washed with cold PBS and harvested. Cell lysates were prepared using RIPA buffer containing 50 mM Tris-HCl (pH), 150 mM NaCl, 1% NP-40, 1 mM DTT, 0.1 mM NaF, 1 mM PMSF, 1 µg/ml pepstatin, 1 µg/ml leupeptin, and 1 µg/ml aprotinin. Protein concentration was determined by the bicinchoninic acid (BCA) method using bovine serum albumin (BSA) as a standard. Equal amounts of protein were separated on 10–12% sodium dodecyl sulfate-polyacrylamide gel electrophoresis (SDS-PAGE) gels. The gels were subsequently transferred onto a polyvinylidene difluoride membrane (Millipore Corporation, Bedford, MA, USA) by electroblotting for 2 h at 60–75 V. The membranes were blocked in a 5% nonfat milk solution in Tris-buffered saline (TBS) with 0.5% Tween-20, and incubated with primary antibodies as indicated. Pre-stained protein markers were used for molecular-weight determination.

### Staining of Autophagosomes with GFP-LC3 and Confocal Microscopy

Approximately 1×10^?5^ cells were seeded in coverglass-bottom-dish, incubated overnight, and then transfected with the adenovirus encoding green fluorescent protein-microtubule-associated protein 1 light chain 3 (AdGFP-LC3; a gift from Dr. J-S Kim at University of Florida) at a concentration of 1,000 virus particles/cell in DMEM. After incubation for 24 h, cells were treated with compounds or starved. For visualization of lysosomes, cells were incubated with LysoTracker® at a concentration of 60 nM for 1 h. Confocal images were obtained with FV10i FLUOVIEW Confocal Microscope (Olympus, Tokyo).

### Homology Modeling of mTOR

A three-dimensional model of mTOR (NCBI GI number : 4826730) comprising 2,549 amino acids was built using SWISS-MODEL program [23] based on homology modeling [24]. The SWISS-MODEL program automatically provides an all-atom model using alignments between the query sequence and known homologous structure. The homologue structure used for mTOR modeling was the X-ray crystallography structure of PI3K (Phosphoinositide 3-kinase) (PDB ID: 1E8X). In particular, the ATP binding site of PI3K is similar to the ATP binding site of mTOR (18% sequence identity and 6.9E-44 e-value) [25]. To validate the quality of predicted structure model, we checked the Z-value using QMEAN server (http://swissmodel.expasy.org/qmean). In order to prepare for docking simulation, we performed deleting solvent, adding hydrogen, adding charge, and replacing incomplete side chain of the structure model using the Chimera program (http://www.cgl.ucsf.edu/chimera/). To add charge, we used the charge data of AMBER ff99SB for standard residues.

### *In silico* Docking of mTOR and Target Compound

For docking simulation, we used the AutoDock 4.2 program according to the software’s manual. Among the many tools available for *in silico* protein-ligand docking, AutoDock4.2 is the most commonly used because of its automated docking capability [26]. To define the docking pocket, we used a set of predefined active sites determined from the structure of PI3K in complex with ATP. We tested docking simulation of mTOR and compound, and ATP using the ATP binding region. To prepare compound for the docking simulation, we performed the following steps: (1) conversion of 2D structure into 3D structure, (2) calculation of charges, and (3) addition of hydrogen atoms using the ChemOffice program (http://www.cambridgesoft.com).

### Statistical Analysis

ANOVA was used to analyze differences among all groups. Differences in the means of individual groups were assessed by Fisher’s protected least significant difference post hoc test. *P* values of <0.05 were considered statistically significant.

## Results

### 1. Increased LC3II Levels and LC3II-positive Vacuoles by MHY1485

First, we evaluated the LC3II/LC3I ratio in normal rat liver Ac2F cells treated with MHY1485 with different doses and times. MHY1485 markedly increased the LC3II/LC3I ratio dose-dependently and time-dependently (Fig. 2A and B). As shown in fig. 2, the LC3II/LC3I ratio was increased due to presumably inhibited LC3II degradation. Next, to monitor the formation of autophagosomes which is characterized by LC3 aggregation, formation of puncta was observed. For this, the cells were treated with the MHY compound after transfection with AdGFP-LC3. In comparison with the control cells that showed the spread of fluorescence and formation of few LC3-puncta, many autophagosomes were seen in the form of perinuclear puncta of GFP-LC3 in the cells treated with our compound after 1 h exposure. However, the size of these LC3-positive vacuoles further increased with longer MHY1485 treatment and finally filled the cytoplasm with vacuoles (Fig. 2 C). Merged images (bottom) show the ring-shaped vacuoles in phase contrast images overlapping with the LC3-positive vacuoles (green).

### 2. Inability of MHY1485 to Increase Autophagic Flux

To confirm that the increased LC3II/LC3I ratio was an outcome of the accumulation of autophagosomes and not elevated autophagic activity, we measured autophagic flux with the lysosome inhibitors bafilomycin A1 and chloroquine [8], [15]. We also presumed that the expansion of the LC3-positive vacuoles occurred because the autophagosomes did not fuse with the lysosomes, which is the final destination of the autophagosome. Bafilomycin A1 or chloroquine was added 1 h before the cell harvest to inhibit lysosomal activity leading to accumulation of LC3II protein. Western blot analysis showed that LC3II protein increased after treatment of bafilomycin A1 or chloroquine, implying the basal autophagic activity. In contrast, MHY1485 treatment increased the ratio of LC3II/LC3I, which was accompanied by no further increase of LC3II by bafilomycin A1 or chloroquine treatment. As our expectation, LC3II protein did not accumulate after treatment with the lysosomotropic reagents in the cells treated with MHY1485, indicating that MHY compound did not increase the autophagic flux and suppressed the basal level of autophagic flux (Fig. 3A and B). Because p62 acts as a scaffolding adaptor protein interacting with both LC3II and polyubiquitinated protein, and degrading with these proteins in autolysosomes, changes in p62 was measured. In the present result, the p62 level did not decrease or slightly increased in MHY1485-treated cells (Fig. 3C). Beclin-1 is required for the initiation of the formation of autophagosomes, and increases in autophagic process. MHY1485 did not change the level of beclin-1 compared to controls (Fig. 3D). Therefore, we determined that MHY1485 inhibited the autophagic process.

### 3. Inhibition of Starvation-induced Autophagic Flux by MHY1485

Next, to confirm the inhibitory effect of MHY1485 on autophagy, we evaluated the autophagic flux and the colocalization of LC3-positive vacuoles and lysosomes using confocal microscopy in cells stimulated by starvation, which is a powerful circumstance leading to autophagy. Active autophagic processes occurred in cells with starvation, as demonstrated by increased accumulation of LC3II protein after treatment of bafilomycin A1 or chloroquine. However, the extent of accumulated LC3II protein was less after treatment of bafilomycin A1 or chloroquine in the MHY1485 treated starved cells (Fig. 4A). These data strongly indicate decreased autophagic flux by MHY1485. Consistent with this, the result using chloroquine, another inhibitor of lysosomal fusion, showed a similar inhibitory effect of MHY1485 on the autophagic flux induced by starvation (Fig. 4B). We assessed the colocalization of autophagosome with lysosomes to confirm the level of their fusion. Ac2F cells were transfected with AdGFP-LC3 and stained with LysoTracker to visualize the lysosomes. LC3 and lysosomal signal overlap in cells under starvation decreased by MHY1485 co-treatment, indicating that starvation-induced fusion between autophagosomes and lysosomes was inhibited by the MHY compound (Fig. 5). The percentage of overlap index was 132.7% in cells with starvation, 127% in cell with starvation plus MHY1485, and 121% in cell treated only MHY1485.

### 4. Increased mTOR Activity and Docking Simulation of MHY1485 with mTOR

mTOR, a major regulator of autophagy, plays a role in the inhibition of autophagy through the phosphorylation of unc-51-like kinase 1 (ULK1), a component involved in the initiation of autophagic process [13]. Recently, lysosomal positioning of mTOR has been identified. mTOR is recruited to the surface of lysosomes where it becomes activated. Morpholino triazine structure, which our compound also possesses, has also been reported to easily bind mTOR and inhibit its activity. Therefore, we examined the effect of our compound on the activity of mTOR. The phosphorylation of mTOR at ser2448, which implies the activated form of mTOR, dose-dependently increased in MHY1485-treated cells (Fig. 6). The level of phosphorylation of eukaryotic-translation initiation factor 4E-binding protein 1 (4E-BP1) which is the typical downstream substrate of mTOR [27] was also increased in a dose-dependent manner. Rapamycin was used as a positive control. To confirm that the MHY compound binds mTOR, the docking simulation of the compound with mTOR was carried out. We checked the Z-score of the 3D structure to validate predicted structure models using the QMEAN server. Because mTOR had low sequence identity (18%) with the template structure (1E8X), the Z-score of the predicted model was −4.8. However, we checked the quality of each residue on the mTOR structure (Fig. 7A). As a result, the internal region that bound ATP and compounds was more reliable (color is blue) than the external region (color is red) of mTOR. The estimated residue error was visualized using a color gradient from blue (more reliable regions) to red (potentially unreliable regions, estimated error above 3.5 Å). The docking simulation was successful with significant scores. The binding energies of the compounds were −7.28 kcal/mol (PP242) and −7.55 kcal/mol (MHY1485). The docking score between the ligand and the receptor is represented by various energy terms such as electrostatic energy, van der Waals energy terms, and the solvation energy (Fig. 7B). MHY1485 showed a higher docking score with mTOR than PP242, a first selective inhibitor that targets the ATP domain of mTOR [28].

## Discussion

Autophagy is a self-degradation process via the lysosomal pathway that is required for the normal turnover of cellular components and the starvation response [1], [2]. The autophagic process involves a unique membrane structure, the autophagosome which is a spherical structure consisting of double bilayer membranes. In the final stage of autophagy, autophagosomes fuse with lysosomes for degradation of contents in the autophagosome [7], [29]. The present study verified that the synthesized compound, MHY1485, inhibited the fusion between autophagosomes and lysosomes leading to accumulation of LC3II protein and enlarged autophagosomes in rat hepatocytes and also induced the activity of mTOR.

It has been reported that autophagic markers such as LC3 and beclin-1 increase when the fusion between autophagosomes and lysosmes is blocked [30], [31]. In our study, treatment with MHY1485 led to the accumulation of LC3II and enlargement of cellular vacuoles. This phenomenon is very similar to that induced by treatment with chloroquine, a typical inhibitor of autophagy. It has been reported that chloroquine-treated human retinal pigment epithelium-derived ARPE-19 cells demonstrated a marked increase in vacuolation and LC3II protein by blocking the lysosomal fusion [11], [32]. While the conspicuous cytosolic vacuoles induced by chloroquine were dilated lysosomes, the MHY1485-induced vacuoles were the enlarged LC3-positive vacuoles shown in Fig. 2 C. Further, it has been speculated that chloroquine is easily accumulated in lysosomes due to its character as a weak base [11], [33]. MHY1485 is also a weak base, but other synthesized compounds that have higher basicity than MHY1485 did not show cellular vacuolation. Therefore, we considered that the MHY1485-induced enlargement of the cellular vacuoles was not caused by its basicity.

Many small molecules that induce the autophagic process including rapamycin, SAHA, temozolomide and curcumin have been explored [34], [35], [36], but few materials have been found to have inhibitory effects on autophagy. The maintenance of autophagy is important for normal cellular conditions, and cancer cells also employ autophagy as a cellular survival pathway. Therefore, pharmacological inhibitors of autophagy have been used with combinations of chemotherapy [37], [38], [39], [40]. The inhibitors of autophagy generally exhibit cellular toxicity and induce cell death. We examined the viability of Ac2F cells treated with MHY1485 for 24 h at different concentrations. MHY1485 concentrations at over 20 µM induced a 20% decline of the cell viability compared to untreated cells. This concentration was 10-fold higher than 2 µM of MHY compound that was sufficient to have an inhibitory effect on autophagy. Treatment of MHY1485 2 µM also did not show any cell death during longer treatment, supporting that MHY1485 had less toxicity than other well-known inhibitors.

One interesting finding is the activation of mTOR by MHY1485. mTOR is a major regulator of initiation of autophagy [13]. The recent identification of mTOR localization at the lysosomal membrane when it is activated [41], [42] and the several reports that morpholino triazine analogs can easily bind mTOR [43], [44] led us to the question about the effect of MHY1485 on the activity of mTOR. At first, we expected that the MHY compound would inhibite mTOR activation, because some morpholino triazine analogs have been identified as a selective ATP-competitive mTOR inhibitor [43], [44] and because on docking simulation, the MHY compound showed the higher docking score than PP242, a first selective inhibitor that targets ATP domain of mTOR [28]. Contrary to our expectation, MHY1485 induced the activation of mTOR as described in the results. Although further study is required to identify the reasons, we speculated 2 possibilities: 1) MHY1485 might bind a different site from an ATP-binding site of mTOR. 2) MHY1485 might indirectly activate mTOR. In respect that the phosphorylated mTOR at ser2448 elevated, there is a possibility of indirect activation on mTOR by the MHY compound. Because the PI3K/Akt signaling pathway phosphorylates mTOR at ser2448 [45], MHY1485 may activate upstream signaling of mTOR. Further study will also be needed to verify an association between MHY1485-induced activation of mTOR and regulation of autophagy.

In conclusion, we found a novel mechanism for inhibition of autophagy by MHY1485. The effects of the synthetic compound MHY1485 included the inhibition of autolysosome formation, leading to the arrest of autophagy and the induction of mTOR activity.
