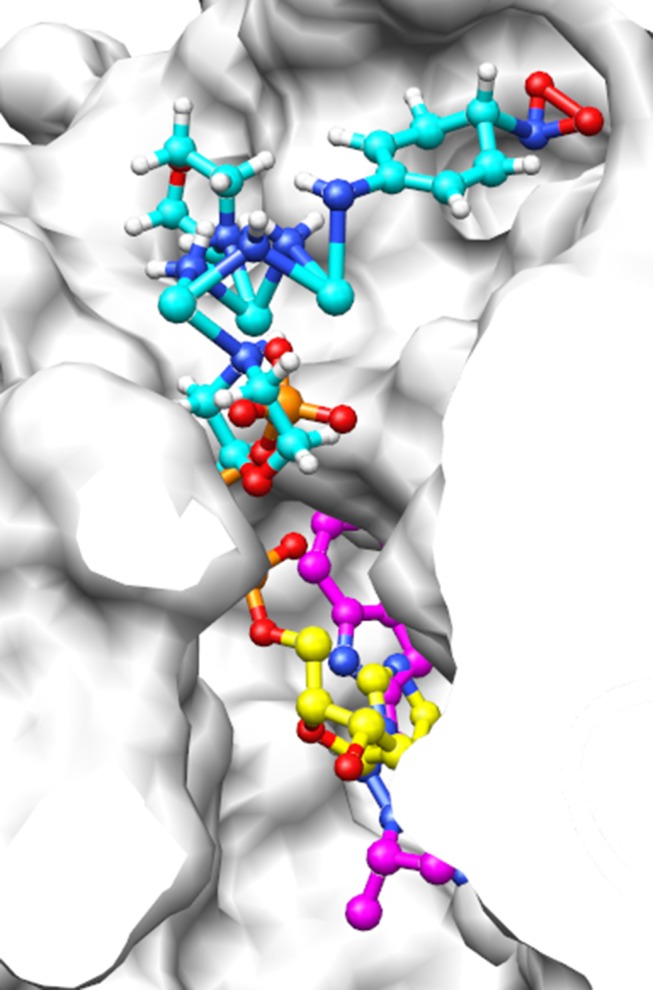# Correction: Inhibitory Effect of mTOR Activator MHY1485 on Autophagy: Suppression of Lysosomal Fusion

**DOI:** 10.1371/annotation/e3163ad5-f3d8-4a21-b3e1-f2033a76f9db

**Published:** 2013-01-29

**Authors:** Yeon Ja Choi, Yun Jung Park, Ji Young Park, Hyoung Oh Jeong, Dae Hyun Kim, Young Mi Ha, Ji Min Kim, Yu Min Song, Hyoung-Sam Heo, Byung Pal Yu, Pusoon Chun, Hyung Ryong Moon, Hae Young Chung

Figure 7 in the article reported a structure that included too many hydrogen atoms on the carbons of the aromatic rings, the authors are therefore providing this revised figure which includes the correct number of hydrogen atoms: 

**Figure:**